# Erratum to: Resolution of ST deviation after myocardial infarction in patients with and without sleep-disordered breathing

**DOI:** 10.1007/s11818-018-0165-5

**Published:** 2018-05-23

**Authors:** Ulrich Sterz, Stefan Buchner, Andrea Hetzenecker, Anna Satzl, Kurt Debl, Andreas Luchner, Oliver Husser, Okka W. Hamer, Claudia Fellner, Florian Zeman, Lars S. Maier, Michael Arzt

**Affiliations:** 1 Klinik und Poliklinik für Innere Medizin II, Universitätsklinikum Regensburg, Franz-Josef-Strauss-Allee 11, 93053 Regensburg, Germany; 2 Medizinische Klinik I, Klinikum Amberg, Amberg, Germany; 3 Deutsches Herzzentrum München, Klinik a.d. Technischen Universität München, Munich, Germany; 4 Institut für Röntgendiagnostik, UniversitätsklinikumRegensburg, Regensburg, Germany; 5 Zentrum für klinische Studien, Universitätsklinikum Regensburg, Regensburg, Germany

**Erratum to:**

**Somnologie 2018**

10.1007/s11818-018-0154-8

The article “Resolution of ST deviation after myocardial infarction in patients with and without sleep-disordered breathing”, written by Ulrich Sterz, Stefan Buchner, Andrea Hetzenecker, Anna Satzl, Kurt Debl, Andreas Luchner, Oliver Husser, Okka W. Hamer, Claudia Fellner, Florian Zeman, Lars S. Maier, Michael Arzt, was originally published electronically on the publisher’s internet portal (currently SpringerLink) on 14 March 2018 without open access.

With the authors’ decision to opt for Open Choice the copyright of the article changed on May 14th 2018 to © The Author(s) [2018] and the article is forthwith distributed under the terms of the Creative Commons Attribution 4.0 International License (http://creativecommons.org/licenses/by/4.0/), which permits use, duplication, adaptation, distribution and reproduction in any medium or format, as long as you give appropriate credit to the original author(s) and the source, provide a link to the Creative Commons license and indicate if changes were made.

